# A Lethal Pursuit of Beauty: Tight-Lacing, the Faja Corset, and a Subcapsular Hematoma

**DOI:** 10.7759/cureus.9825

**Published:** 2020-08-18

**Authors:** Jayaram Kumaraswamy, Joshua Levy, Randoll Christopher

**Affiliations:** 1 Internal Medicine, Philadelphia College of Osteopathic Medicine, Philadelphia, USA; 2 Medicine, Philadelphia College of Osteopathic Medicine, Philadelphia, USA; 3 Surgery, Mercy Catholic Medical Center, Philadelphia, USA

**Keywords:** liver hematoma, faja corset

## Abstract

Faja corset, a tight garment worn to obtain an hourglass body, may have contributed to the formation of a spontaneous subcapsular hematoma in a 38-year-old nulligravid woman with no history of trauma. Subcapsular hematomas of the liver are a rare occurrence, with many documented cases arising during pregnancy as a complication of HELLP (hemolysis, elevated liver enzymes, and low platelet) syndrome. Additional causes include iatrogenic trauma, rupture of cysts, hepatic adenomas, hepatocellular carcinoma, or coagulopathies. Idiopathic or spontaneous cases are very rare. In this report, we discuss the case of a 38-year-old female who presented with a spontaneous subcapsular liver hematoma of unclear etiology, most likely related to compression by her faja corset.

## Introduction

*Faja *is a Spanish word that means "wrap." A faja is essentially a corset that is used by women to obtain an hourglass figure, a phenomenon known as tight-lacing [1]. Modern America has historically frowned upon such garments, as they can be painful to the body. In the past decade, however, corsets have become more accepted, especially in the Latin American community. The compressive forces induced by wearing a faja corset may have contributed to the formation of a subcapsular liver hematoma in our patient, similar to a phenomenon known as tight-laced liver. Here we discuss a patient with no identifiable etiology other than her recent decision to wear a tight faja corset, which most likely contributed to the formation of her subcapsular liver hematoma.

## Case presentation

**Figure 1:**
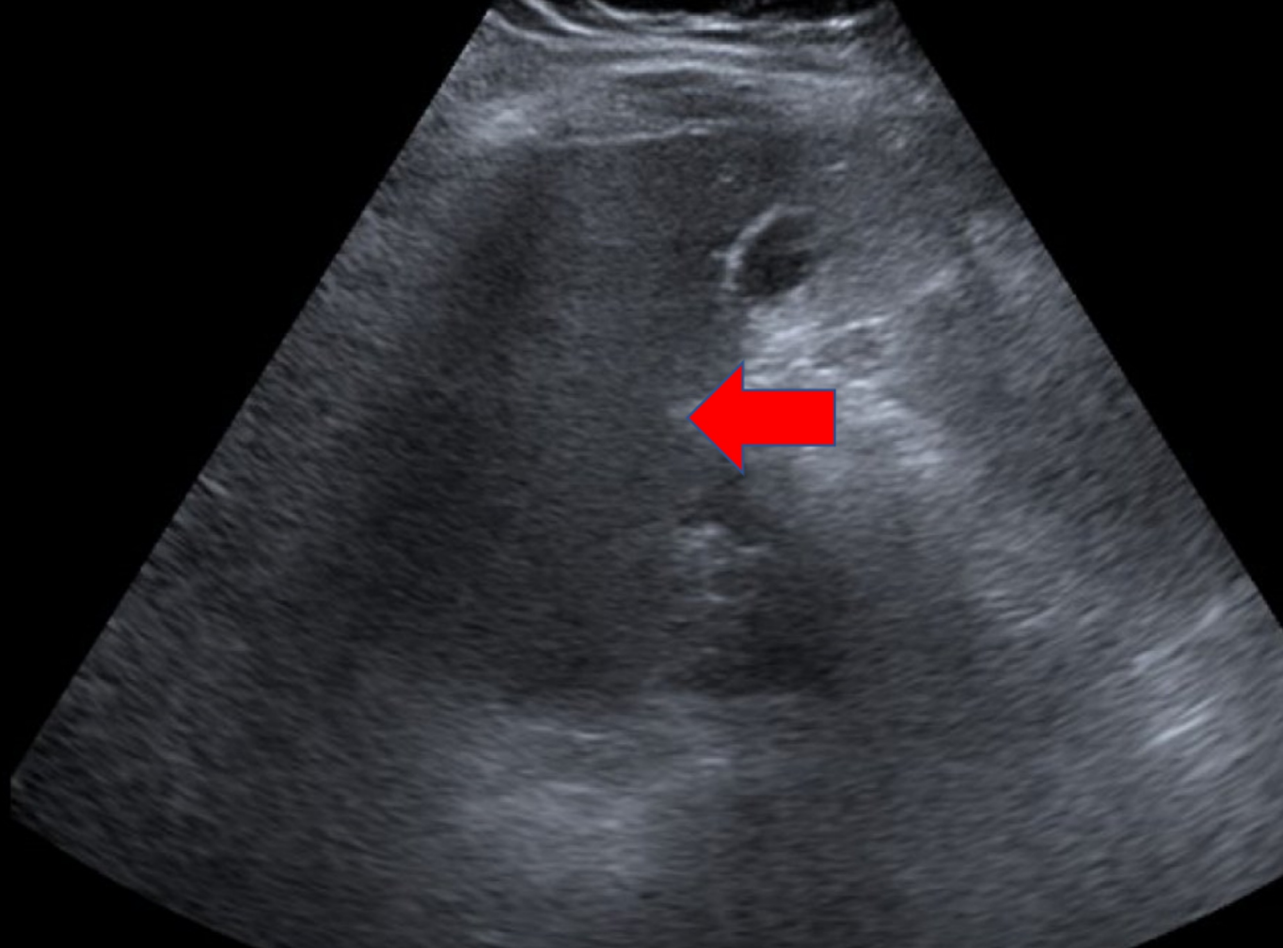
Ultrasonography showing a fluid collection in the liver

**Figure 2:**
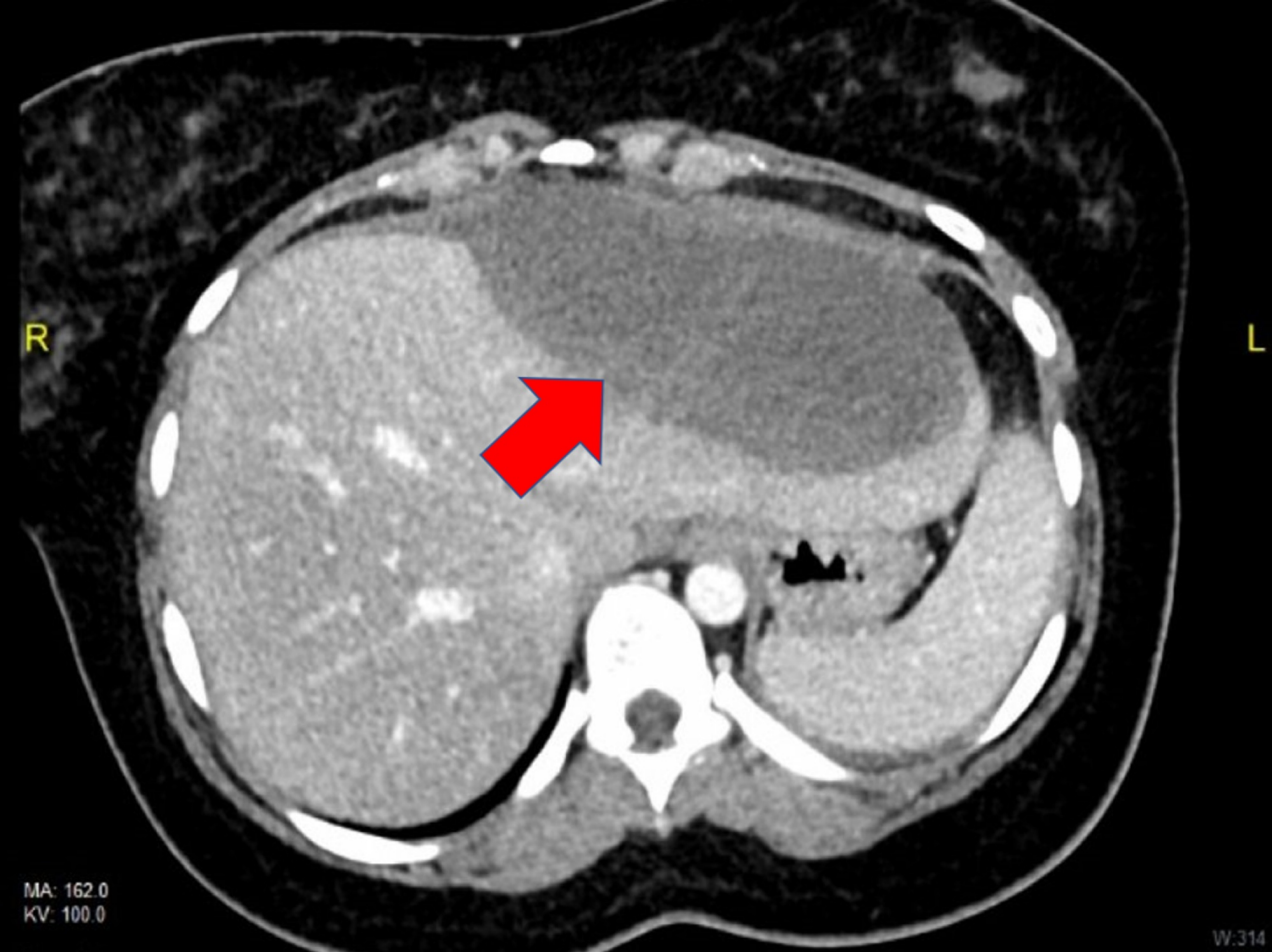
An abdominal CT scan showing a 13.7 x 5.6 x 9.5 cm hepatic subcapsular hematoma

A 36-year-old G0P0 Hispanic female with no significant past medical history presented to the emergency room with unremitting sharp epigastric pain radiating to her right side and back of four days' duration. The pain began suddenly with no precipitating event, was constant, and was unrelieved by changes in position. Nonsteroidal anti-inflammatory drugs (NSAIDs) provided minimal relief. Prior to the sudden onset of these symptoms, the patient had begun to wear a faja corset every day, taking it off only occasionally to shower. Review of systems was otherwise negative, and no associated symptoms were reported. The patient reported drinking less than two alcoholic beverages per week, and denied smoking and IV drug use. No significant past medical history was noted. Vital signs were stable, with oxygen saturation 98% on room air. Physical examination was notable for epigastric and right upper quadrant (RUQ) tenderness. Bowel sounds were auscultated. The abdomen was nondistended and without rebound, guarding, or rigidity. The remainder of the physical exam was unrevealing. Complete blood count (CBC), renal function, liver enzymes, amylase, and lipase were within normal limits. A pregnancy test was negative. Abdominal ultrasound revealed hepatomegaly and a fluid collection on the anterior aspect of the left hepatic lobe, consistent with a hematoma (Figure 1). CT of the abdomen/pelvis confirmed a 13.7 x 5.6 x 9.5 cm subcapsular hematoma (Figure 2). With these findings, a diagnosis of subcapsular hematoma of the liver was given. Initially the patient was made nil per os (NPO), with a surgery consultation in place. Surgery suggested that surgical intervention was not recommended at the time as the patient was hemodynamically stable. Additional management included analgesic pain control and muscle relaxants. The patient remained hemodynamically stable from admission to discharge, and required no blood transfusion. Six days from admission, the patient was discharged home with resolution of her pain and was advised to stop wearing faja corsets.

## Discussion

Subcapsular hematomas of the liver represent an expansion of blood between Glisson’s capsule and the hepatic parenchyma [2]. Idiopathic and spontaneous occurrence is exceedingly rare [3]. Hematoma formation is often linked to Iatrogenic injury from endoscopic retrograde cholangiopancreatography (ERCP), hepatobiliary surgery, and liver biopsy. Additionally, underlying liver pathologies, such as HELLP (hemolysis, elevated liver enzymes, and low platelet) syndrome, hepatocellular carcinoma, hepatic adenomas, and focal nodular lesions, have been linked to hematoma formation [4]. In the case of the patient described above, no iatrogenic trauma or underlying liver pathology was noted. Given the patient’s recent decision to wear a faja corset, a practice known as tight-lacing, and subsequent formation of the subcapsular liver hematoma, we propose that compression, bruising, and possible ischemia of the liver were caused by the corset, resulting in hematoma formation. 

The negative effects of corsets have been documented since the 1800s, with 19th century physicians noting pulmonary distress and internal organ damage of their patients [5]. A phenomenon known as tight-laced liver has also been well described, with women who wear tight corsets presenting with RUQ pain secondary to cholestasis and dyspepsia [6]. The cholestasis was attributed to the compressive damage caused by corsets. The pathogenesis of the patient’s hematoma therefore seems to be directly related to the faja corset or tight-lacing. 

Once the hematoma has formed, distension of the liver capsule will cause RUQ pain. The primary concern of subcapsular liver hematomas is lethal hemorrhage [3]. Nonoperative management is preferred unless the patient is hemodynamically unstable or severe trauma has occurred [2]. In the case of the hemodynamically stable patient described above, conservative management was provided, managing pain, and continuous monitoring for hemodynamic changes. 

## Conclusions

A once old and thought to be outdated form of beautification, tight-lacing has resurfaced as a popular way to obtain an hourglass body shape. Hepatic and biliary injury may be attributable to this practice, which is achieved by wearing tight corsets, termed faja in the Hispanic community. The idea that these corsets cause internal organ damage was documented hundreds of years ago, but recent literature on the topic is not available, as the popularity of tight-lacing declined in the mid-1900s. In the presence of no underlying liver pathology or iatrogenic trauma, constricting garments may contribute to the formation of subcapsular hematomas and other internal organ pathologies by processes of compression, bruising, and ischemia. Newfound popularity of corsets and tight-lacing should warrant new investigation on long-term damage caused by using such devices.
